# Bioaccumulation of Heavy Metals and Metalloids in Tissues of Wild Boar (*Sus scrofa*) in the Campania Region, Southern Italy: A Pilot Study

**DOI:** 10.3390/toxics14070626

**Published:** 2026-07-17

**Authors:** Sara Damiano, Francesca Paola Nocera, Consiglia Longobardi, Valeria Iervolino, Carlo Romei, Angela Amoresano, Carolina Fontanarosa, Gennaro Battaglia, Rossana Schena, Annunziata Romano, Nadia Piscopo, Antonio Raffaele, Giuseppe Rizzo, Serena Montagnaro, Roberto Ciarcia

**Affiliations:** 1 Department of Veterinary Medicine and Animal Productions, University of Naples “Federico II”, 80137 Naples, Italynadia.piscopo@unina.it (N.P.); roberto.ciarcia@unina.it (C.R.); 2 Department of Chemical Sciences, University of Naples “Federico II”, 80137 Naples, Italy; 3 Consorzio Interuniversitario Istituto Nazionale Biostrutture e Biosistemi-INBB, Via dei Carpegna 19, 80165 Rome, Italy; 4 Local Management Hunting Authority (ATC), Province of Avellino, Campania Region, 83100 Avellino, Italy

**Keywords:** heavy metals, wild boar, liver, muscle, testicle

## Abstract

Wild boars (*Sus scrofa*) are reliable bioindicator species for assessing environmental pollution. In this pilot study, we measured concentrations of arsenic (As), cadmium (Cd), copper (Cu), selenium (Se), zinc (Zn), and lead (Pb) in liver, diaphragm, and testicular tissues from wild boars collected across seven districts of Avellino province (liver/diaphragm: *n* = 87; testicles: *n* = 50). Pb reached its highest median value in the diaphragm (0.43 mg/kg w.w.; maximum 14.80 mg/kg), while Cd showed a pronounced affinity for hepatic tissue (median 0.15 mg/kg; maximum 0.62 mg/kg). Zn was the most abundant element overall, particularly in the diaphragm (median 20.51 mg/kg; maximum 46.81 mg/kg). Se concentrations were generally below the detection limit (<0.001 mg/kg), with only occasional outliers. Sex-related differences were evident: males accumulated more Pb in the liver, while females had higher levels of Cd and As. Spatial patterns identified AVMFS009 and AVAR009 as hotspots for Cu, Zn, and Cd, while AVCP003 emerged as the main hotspot for As. Overall, the results reinforce the role of wild boars as effective bioindicators in southern Italy.

## 1. Introduction

Environmental pollutants, such as heavy metals and metalloids, are a global problem due to their persistence, resistance to degradation, and accumulation and biomagnification in food chains, including drinking water [1,2]. Cadmium (Cd), lead (Pb), and arsenic (As) are common environmental contaminants that may be present naturally due to volcanic activity and weathering of rocks, or from anthropogenic sources such as industrial emissions, agricultural activities, and poor waste disposal [3]. Once released into the environment, they can persist for long periods, leading to continual exposure and uptake by plants and animals. This paper discusses essential trace elements and non-essential toxic metals, which have distinct biological activities and toxicological properties [4]. Copper (Cu) and zinc (Zn) are necessary minerals involved in important functions, including enzymatic activity, antioxidant defense, and cell metabolism, but can cause oxidative damage and tissue toxicity when excessively accumulated [5,6]. Selenium (Se) is an essential trace element for redox regulation and immune function; however, the margin between its physiological requirement and toxicity is small, and it can be hazardous when homeostasis is disrupted [6,7]. In contrast to essential elements, non-essential heavy metals such as Cd and Pb have no known physiological role and are highly toxic even at low concentrations [8,9]. Cd is one of the most toxic heavy metals and is associated with environmental and industrial pollution. It is released from many anthropogenic sources, such as fossil fuel combustion, agricultural runoff, landfill leachates, electroplating, and the manufacturing of Ni–Cd batteries, pigments, stabilizers, and alloys. Cd is recognized internationally as a pollutant and is listed in the International Register of Potentially Toxic Chemicals by the United Nations Environment Programme [10,11] due to its well-documented carcinogenic and mutagenic effects. Pb is a contaminant of concern for animal and public health due to its well-known adverse effects, including neurological and reproductive issues [12,13]. In animals, it can cause sudden death if fragments of Pb ammunition remain in the body. Ingestion of small amounts of Pb often results in various clinical effects, including encephalopathy, gastroenteritis, and peripheral nerve degeneration [14]. As is a well-known poisonous metalloid that, due to industrial, mining, and agricultural activities that damage the environment, bioaccumulates in wildlife such as wild boar. Wild boars’ rooting behavior exposes them to contaminated plant and soil substrates resulting from previous pesticide applications and As discharge into soils and water systems by chemical and metallurgical industries (active or abandoned) [15]. This exposure leads to prolonged bioaccumulation, primarily in liver, kidney, and muscle tissues [16]. The wild pig (*Sus scrofa*) is a good sentinel species for monitoring environmental contamination because it is widely distributed (including urban and suburban regions), omnivorous, and closely associated with both natural and anthropogenic landscapes [17,18,19]. Consequently, this ungulate can bioaccumulate contaminants from soil, water, and food, making it a promising candidate for monitoring the presence and mobility of harmful substances in terrestrial environments, especially in areas with overlapping human activity, natural geochemical variation, and complex exposure scenarios. Among these is the Campania region in Southern Italy, with its diverse land use, industrial and agricultural demands, waste management problems, and volcanic substrates [20]. The Campania region is a dynamic area for veterinary public health and wildlife monitoring, where retrospective baseline data are essential for tracking health and environmental trends in animal populations [21]. This scenario includes several areas of the Avellino province, previously recognized as potential pollution hotspots where environmental matrices can serve as reservoirs of heavy metals and metalloids. Understanding the toxicokinetics and bioaccumulation pathways of potentially toxic elements (PTEs) in wildlife in these areas is important for assessing ecosystem health and conducting preliminary compliance screening against European meat standards.

To achieve a comprehensive toxicological assessment, a multi-matrix approach is essential. Organs such as the liver and skeletal muscle (including the diaphragm) are conventional matrices for monitoring overall body burden and evaluating compliance with established contaminant thresholds. However, including reproductive tissues provides unique insight into the effects of chronic exposure on reproductive functions. By examining testicular bioaccumulation, this study aims to evaluate potential reproductive hazards in wild fauna and assess the functional integrity of the blood-testis barrier (BTB) under natural exposure conditions. The present study aimed to investigate the presence and spatial distribution of certain heavy metals and metalloids in wild boars hunted in different areas of the province of Avellino. The contents of Cu, Zn, As, Se, Cd, and Pb were analyzed in diaphragm, liver, and testicular tissues using established analytical techniques. These elements were selected based on their toxicological relevance and importance for food safety standards [22]. The combination of tissue-specific accumulation patterns with geographic information is likely to increase knowledge of environmental exposure pathways and will aid further monitoring in this area of considerable ecological and public health significance.

## 2. Materials and Methods

### 2.1. Ethics Statement

For this study, approval from the Ethics Committee was not required, as no live animals were handled and no specimens were killed specifically for research purposes. Biological samples were collected postmortem through convenience sampling, using organs voluntarily provided by licensed hunters from free-ranging wild boars legally harvested during official hunting seasons. All hunting activities strictly complied with the 2025–2026 annual wildlife management and culling plan (hunting season from 1 October 2025 to 31 January 2026), approved by the competent local authorities of Avellino Province in the Campania Region, Italy.

### 2.2. Study Area and Sample Collection

**Figure 1:**
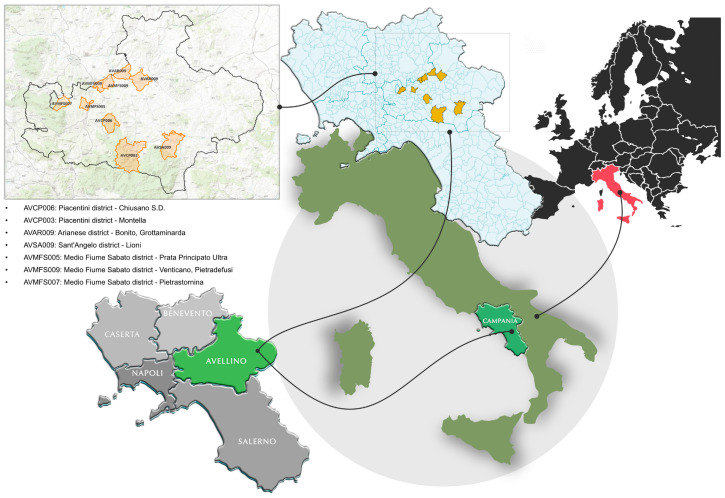
Cartographic representation of the province of Avellino (Campania region, southern Italy) showing hunting zones (in orange) designated for wild boar (*Sus scrofa*) sampling. The map was created with Affinity Designer 2, version 2.6.3.3322, Serif (Europe) Ltd., Nottingham, UK.

This study focused on free-ranging wild boars (*Sus scrofa*) harvested in the province of Avellino, located in the Campania Region of Southern Italy. The Avellino province features highly varied topography, including hilly terrain and lowlands that significantly influence the spatial distribution and population dynamics of wild boars. Geographically centered at approximately 41° N, 14° E, the area exhibits substantial altitudinal variation, ranging from 300 m a.s.l. in the lower valleys to over 1400 m a.s.l. in prominent mountainous formations such as the peaks of Monte Terminio and Monte Partenio. The province covers about 28,000 hectares, with complex limestone mountains, dense deciduous woodlands, and interspersed agricultural areas, collectively providing a highly suitable ecological niche for this ungulate species. The typical Mediterranean climate, with warm summers and humid winters, ensures stable trophic resources—including acorns, tubers, hypogeous fungi, and cultivated crops—that further support population growth. Sampling was conducted during regular hunting seasons in designated hunting areas within the province of Avellino. The specific districts involved were AVAR009 (Bonito/Grottaminarda), AVCP006 (Chiusano S.D.), AVSA009 (Lioni), AVCP003 (Montella), AVMFS007 (Pietrastornina), AVMFS005 (Prata P.U.), and AVMFS009 (Venticano/Pietradefusi). The hunting areas included in this study are shown in Figure 1.

A total of 87 wild boars were sampled, including 37 females and 50 males, with body weights ranging from 30 to 160 kg. Biometric data, including sex and total weight, were recorded for each specimen upon collection. The age of animals above one year was determined using the tooth eruption method [23]. Based on estimated chronological age and dental development, wild boar specimens were assigned to three biological age classes to evaluate age-dependent bioaccumulation patterns: (i) Young (≤1.5 years, including piglets and young subadults; *n* = 33); (ii) Adult (1.5–5 years, sexually mature, actively reproducing individuals; *n* = 46); and (iii) Senior (>5 years, older specimens with prolonged environmental exposure; *n* = 8). Individual body weight was recorded for all specimens as a continuous proxy for growth. From each animal, representative aliquots of fresh liver, diaphragm, and testicular tissues (the latter collected only from male specimens, *n* = 50) were obtained. Individual tissue samples were immediately placed in sterile polypropylene tubes, transported under refrigerated conditions, and stored at −20 °C until laboratory analysis. The determination of heavy metal and metalloid (As, Cd, Cu, Se, Zn, and Pb) concentrations was performed at the Laboratory of the Department of Chemical Sciences, University of Naples Federico II.

### 2.3. Essential and Non-Essential Elements Analysis with ICP-MS

Elemental analyses were performed using an Agilent Technologies Agilent 7700 ICP-MS (Santa Clara, CA, USA) equipped with a frequency-matching radio frequency (RF) generator and a third-generation Octapole Reaction System (ORS3) operating in helium collision mode. Instrumental operating conditions were as follows: RF power, 1550 W; plasma gas flow rate, 14 L min^−1^; carrier gas flow rate, 0.99 L min^−1^; and helium gas flow rate, 4.5 mL min^−1^. A multielement internal standard solution containing Li, Sc, Ge, Rh, In, Tb, Lu, and Bi was used for signal correction and instrumental drift compensation throughout the analyses.

### 2.4. Preparation of Standard Solutions and Method Validation

Method validation was performed to ensure the accuracy, precision, linearity, and sensitivity of the ICP-MS analytical procedure. The limits of detection (LOD) and limits of quantification (LOQ) for each element were calculated using the blank determination approach, defined as 3σ/*b* and 10σ/*b*, respectively, where σ represents the standard deviation of the procedural blanks (*n* = 10) and *b* is the slope of the calibration curve. Method accuracy (recovery) was evaluated through spiking experiments (sample fortification) at a concentration level of 50 µg L^−1^ (50 ppb). Precision was assessed as the relative standard deviation (RSD, %) of six independent replicates of spiked samples (*n* = 6). The validation parameters, including the correlation coefficients (R^2^) of the calibration curves, LOD, LOQ, recovery rates, and precision for all analyzed elements, are summarized in Table 1.

### 2.5. Sample Treatment

Approximately 0.5 g of homogenized sample was accurately weighed into PTFE vessels. Samples were subjected to wet acid digestion using a mixture of nitric acid and hydrochloric acid (1:3, *v*/*v*). The digestion procedure was carried out at 90 °C for 16 h. After digestion, the resulting solutions were quantitatively transferred into 25 mL volumetric flasks and diluted to volume with ultrapure water. All samples were prepared and analyzed in duplicate. Procedural blanks were processed simultaneously to evaluate potential contamination during sample preparation and analysis.

### 2.6. Statistical Analysis

Statistical analysis was conducted using JMP Student Edition software (Version 17.0, SAS Institute Inc., Cary, NC, USA). The dataset is non-parametric; therefore, values are reported as medians and ranges (minimum–maximum) for each variable.

Normality was assessed with the Shapiro–Wilk test. Nonparametric statistical methods were applied because several analytes were not normally distributed and some had left-censored data (values below the limit of detection, LOD). For trace elements with a high censoring rate (>50%, such as Se and As in most tissues), descriptive statistics are reported as <LOD, and accumulation patterns are discussed based on detection frequencies rather than calculated numerical medians.

Due to the high rate of left-censoring (values below the limit of detection, LOD) observed for arsenic (As) and selenium (Se), exceeding 40% in several subgroups and reaching 93–96% in specific matrices, these two elements were excluded from the multivariate models to avoid statistical artifacts and substitution bias. Consequently, multivariate associations were analyzed using Principal Component Analysis (PCA), focusing strictly on fully quantified elements (Pb, Cd, Cu, and Zn). For univariate descriptive statistics only, values below the LOD were imputed using the LOD/√2 substitution method (0.000707 mg/kg), which remains robust for baseline descriptors when the overall proportion of non-detects does not distort the underlying distribution.

The Kruskal–Wallis omnibus test was used to assess differences in trace element concentrations among the three tissue regions (liver, diaphragm, and testicles) and the sampling locations (AVAR009, AVCP006, AVSA009, AVCP003, AVMFS007, AVMFS005 and AVMFS009). The Steel–Dwass test was used as the post hoc method for pairwise multiple comparisons (*p* < 0.05) following global significance. This test was chosen for its robustness in controlling the familywise Type I error rate under unbalanced and nonhomoscedastic data. Positive differences (excesses) in paired comparisons of geographical areas indicated the sites with the highest accumulation levels.

Differences in trace element concentrations between sexes within the same tissue locations (liver and diaphragm only) were compared using the Wilcoxon–Mann–Whitney test for independent samples. The predetermined significance level was α = 0.05. The testicular matrix was excluded from the analysis of sexual dimorphism since there is no anatomical equivalent in females.

To assess the influence of demographic and physiological factors on heavy metal bioaccumulation while avoiding collinearity, animal age was treated as a categorical factor with three predefined classes (Young, Adult, and Senior). Differences in trace element concentrations (Pb, Cd, Cu, and Zn) across age classes and target tissues (liver and diaphragm) were evaluated using multivariate analysis of variance (MANOVA), followed by nonparametric univariate post hoc tests (Kruskal–Wallis test with Dunn’s multiple comparison test) to identify specific group differences. In parallel, the continuous relationship between metal levels and body weight was assessed using Spearman’s rank correlation (ρ).

To evaluate the continuous relationship between tissue trace element concentrations and individual physiological development, Spearman’s rank correlation coefficient (ρ) was calculated using individual body weight as a continuous proxy for physical growth. This nonparametric correlation analysis was also used to investigate inter-element relationships, identifying potential synergistic accumulation patterns or shared environmental exposure pathways. The analysis was performed across all three target matrices (liver, diaphragm, and testicles); for testicular tissue, the dataset was strictly limited to male specimens (*n* = 50). Correlation coefficients (ρ) were considered statistically significant at alpha = 0.05.

The nonparametric Spearman’s rank correlation coefficient (ρ) was used to determine monotonic relationships between the concentrations of the investigated trace elements (Pb, Zn, Cu, Cd, As, and Se), regardless of data distribution or the presence of outliers. Correlation analysis for each tissue region (liver, diaphragm, and testicles) revealed organ-specific association patterns. Heatmaps were used to display the strength of associations derived from ρ values. Multivariate associations were analyzed using Principal Component Analysis (PCA). Data were centered and scaled to compensate for differences in trace element concentration scales. PCA of the correlation matrix revealed hidden patterns of accumulation and tissue-specific compartmentalization (liver, diaphragm, and testicles). Principal components were selected based on total explained variance and the eigenvalue criterion (>1). Results were presented as a score plot for sample grouping and a loading plot for the contribution of each trace element to the components. The *p*-values of all inferential tests indicated the following statistical significance: *p* > 0.05 ns; *p* < 0.05 *; *p* < 0.01 **; *p* < 0.001 ***.

## 3. Results

Table 2 presents descriptive data on the accumulation levels of heavy metals (Pb, Cd, As) and essential elements (Cu, Zn, Se) in three biological matrices: liver, diaphragm, and testicles. Pb distribution was variable, with the highest median concentration in diaphragm muscle tissue (0.43 mg/kg w.w.) compared to the liver (0.34 mg/kg w.w.). The diaphragm showed large inter-individual differences, with values reaching up to 14.80 mg/kg w.w., which may reflect acute exposures or differences in metal sequestration metabolism. The lowest concentrations were found in testicular tissue (median 0.25 mg/kg w.w.). Cd showed high hepatic tropism, with median liver concentrations (0.15 mg/kg w.w.) substantially higher than those in testicles and diaphragm (0.03 mg/kg w.w.). Zn was the predominant element in all matrices, with the highest values in the diaphragm (median 20.51 mg/kg w.w.), possibly due to the key physiological role this metal plays in muscle performance; the liver and testicular tissues had medians of 16.81 mg/kg w.w. and 11 mg/kg w.w., respectively. Cu content in the liver was much higher (median 2.58 mg/kg w.w.) than in muscle and testicular tissue. Analytical results showed that As and Se concentrations were frequently below the instrumental detection limit. In female wild boars, Se was undetectable (<0.001 mg/kg) in 93.5% of liver samples and 96.7% of diaphragm samples, resulting in a median value of <LOD for both tissues. A similarly high proportion of censored data was observed for As. However, positive outlier samples were found in specific sub-areas: for example, individual male specimens from the AVMFS009 area showed quantifiable hepatic Se levels up to 0.195 mg/kg and diaphragm levels up to 1.100 mg/kg, indicating sharp, localized variations in dietary intake or environmental availability.

Pb showed a heterogeneous distribution, with the highest median value in the diaphragm muscle tissue (0.43 mg/kg w.w.) compared to the liver (0.34 mg/kg w.w.). In the diaphragm, pronounced inter-individual variability was observed, with values reaching up to 14.80 mg/kg w.w., suggesting possible acute exposure or metabolic differences in metal sequestration. Testicular tissue was least affected (median: 0.25 mg/kg w.w.). Cd showed a marked hepatic tropism, with median concentrations in the liver (0.15 mg/kg w.w.) significantly higher than those in the testicles (0.031 mg/kg w.w.) and diaphragm (0.03 mg/kg w.w.). As shown, low accumulation levels, with medians close to or at the detection limit (<LOD) in the diaphragm and testicles, while slightly higher values (0.03 mg/kg w.w.) were found in the liver. Zn was the most abundant element in all matrices analysed, with the diaphragm showing the highest concentrations (median: 20.51 mg/kg w.w.), likely due to the critical physiological role this metal plays in muscle function. The liver and testicular tissue had medians of 16.81 mg/kg w.w. and 11 mg/kg w.w., respectively. Cu levels in the liver were significantly higher than those in the muscle and testicular tissue, with a median of 2.58 mg/kg w.w. Se showed extremely low concentrations in all monitored tissues, with uniform medians of <LOD, indicating that most samples had levels below the analytical sensitivity threshold, despite sporadic outliers in the diaphragm (up to 1.20 mg/kg w.w.).

Table 3 shows the results of the Kruskal–Wallis (χ^2^) test and Steel-Dwass post hoc comparisons between the different biological matrices.

The presence of sexual dimorphism in analyte accumulation was assessed by comparing quantities in males (*n* = 50) and females (*n* = 37) within the same tissue matrices (liver and diaphragm) (Table 4).

Because the data were non-parametric, the Wilcoxon–Mann–Whitney test was used for independent samples. The gonadal region was excluded from this study because no anatomical equivalent exists in females. Results indicated statistically significant sex-dependent differences in tissue accumulation. The Pb concentration in the liver was substantially higher in males than in females (0.43 vs. 0.24 mg/kg; *p* = 0.0002), while Cd (0.22 vs. 0.11 mg/kg; *p* = 0.0048) and As (0.051 vs. <0.001 mg/kg; *p* = 0.0144) levels were higher in females. In the liver, no significant differences were observed for Cu, Zn, or Se (*p* > 0.05). In the diaphragm, the accumulation of several analytes was significantly higher in males than in females (Cd: 0.034 vs. 0.015 mg/kg, *p* = 0.0017; Zn: 21.88 vs. 18.77 mg/kg, *p* = 0.0142; Se: 0.081 vs. <0.001 mg/kg, *p* = 0.0097; As: 0.0195 vs. <0.001 mg/kg, *p* = 0.035). No differences between sexes were found for Pb or Cu in this tissue (*p* > 0.05).

The concentrations of heavy metals and trace elements across the three biological age classes are summarized in Table 5. Statistical analysis showed that biological age had a significant overall effect only on hepatic cadmium (Cd) concentrations (Kruskal–Wallis χ^2^ = 7.53, df = 2, *p* = 0.0231). However, conservative pairwise comparisons performed with the nonparametric Steel–Dwass post hoc test did not reveal any statistically significant differences between specific age groups (*p* > 0.05 for all pairs). No age-related differences were detected for diaphragmatic Cd levels (χ^2^ = 0.70, df = 2, *p* = 0.7059). Similarly, diaphragmatic and hepatic concentrations of lead (Pb), copper (Cu), and zinc (Zn) remained stable and statistically comparable across all age categories investigated (*p* > 0.05). Given the statistical limitations, the overall effect observed for hepatic Cd should be interpreted strictly as a tentative descriptive trend, rather than as conclusive evidence of age-dependent bioaccumulation in the wild boar population under study.

The tissue-specific Spearman’s rank correlation matrices cross-referencing individual body weight and trace element concentrations are presented in Table 6. The correlation analysis revealed distinct bioaccumulation kinetics in relation to animal growth. In the diaphragm, individual body weight showed a significant positive correlation only with Zn concentrations (ρ = 0.2308, *p* < 0.05), while no significant weight-dependent trends were observed for the other elements. In hepatic tissue, trace element accumulation appeared completely independent of physical growth, as no heavy metals exhibited significant correlations with body weight (*p* > 0.05). Conversely, a significant and strong negative correlation was detected between body weight and Cd levels in testicular tissue (ρ = −0.3442, *p* < 0.05). No other metals showed linear trends associated with weight in the gonads.

Regarding inter-element relationships, notable tissue-specific synergistic and antagonistic co-variations were observed. Highly significant positive correlations between the essential elements Cu and Zn were consistently found across all three matrices: diaphragm (ρ = 0.5469, *p* < 0.001), liver (ρ = 0.4251, *p* < 0.001), and testicles (ρ = 0.7564, *p* < 0.001). In the diaphragm, toxic elements co-varied significantly, as demonstrated by the positive correlations of Pb with Cd (ρ = 0.2911, *p* < 0.01) and Cu (ρ = 0.3122, *p* < 0.01). In the liver, Cd levels showed significant positive correlations with both Cu (ρ = 0.2408, *p* < 0.05) and Zn (ρ = 0.2265, *p* < 0.05). Finally, testicular tissue displayed a unique and highly significant negative correlation between Pb and Zn concentrations (ρ = −0.3709, *p* < 0.01).

**Figure 2:**
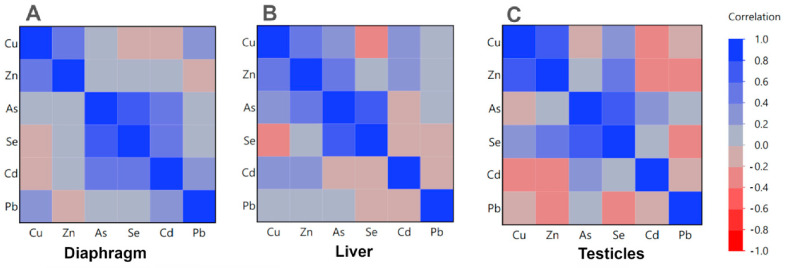
Figure showing Spearman rank correlation heatmaps for trace element concentrations in different tissue types (diaphragm, liver, testicles). The three maps display the Spearman correlation coefficient (ρ) between trace elements (Pb, Cd, As, Cu, Zn, Se) in the diaphragm (**A**), liver (**B**), and testicles (**C**). Correlation values are represented by colors ranging from deep blue (positive correlation, ρ = 1.0) to deep red (negative correlation, ρ = −1.0). Gray or neutral colors indicate no correlation (ρ ≈ 0.0). For selenium (Se) and arsenic (As), correlations were calculated based on ranks, with values below the limit of detection (<LOD) assigned to the lowest shared rank (LOD/√2), ensuring statistical robustness despite censored data. Statistical significance was defined as *p* < 0.05.

Monotonic relationships and correlation patterns among the investigated trace elements in each tissue matrix were confirmed by Spearman rank correlation coefficient analysis (Figure 2).

**Figure 3:**
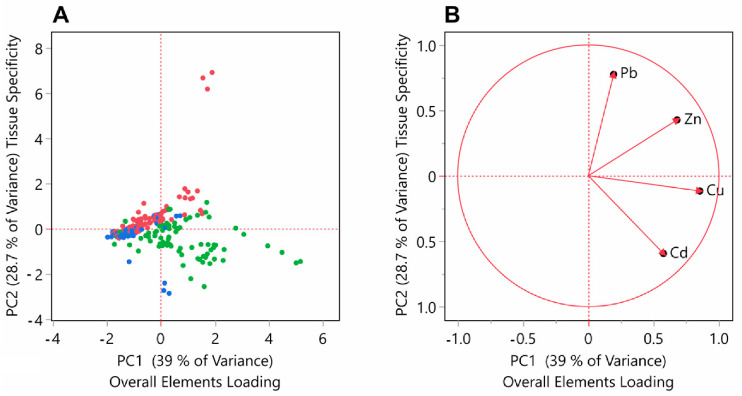
Principal Component Analysis (PCA) based on trace element concentrations in different tissues. (**A**) Score Plot: Spatial distribution of samples (*n* = 224). The first two principal components together explain 67.7% of the total variance (PC1: 39%; PC2: 28.7%). Colors indicate the tissue analyzed: liver (green), diaphragm (red), and testicles (blue). A clear separation is observed along the PC2 axis between metabolic tissue (liver) and muscle tissue (diaphragm). (**B**) Loading plot: vector plot showing the contribution of each quantified element to defining the multivariate space. Cd, Zn, and Cu are the main drivers along the PC1 axis, highlighting a strong co-accumulation pattern, while the vertical orientation of the Pb vector along PC2 reflects its independent bioaccumulation profile, specifically separating the distinct tissue accumulation matrices.

The multivariate relationships among the fully quantified trace elements (Pb, Cd, Cu, and Zn) were evaluated using principal component analysis (PCA). The first two principal components accounted for 67.7% of the total variance (39.0% for PC1 and 28.7% for PC2). As shown in the loading plot (Figure 3B), PC1 was strongly characterized by positive loadings of Cd, Zn, and Cu, indicating a pronounced co-accumulation pattern or shared metabolic pathways for these elements. In contrast, PC2 was almost exclusively driven by the loading of Pb, suggesting that exposure and bioaccumulation dynamics in the sampled wild boar occur independently of the other monitored metals.

**Figure 4:**
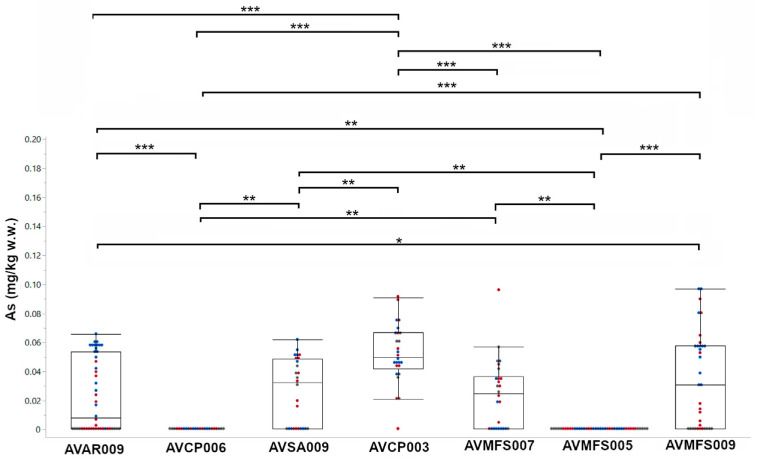
Box-and-whisker plots showing the geographical distribution of As concentrations (mg/kg w.w.) at sample locations in the province of Avellino. The median is represented by a horizontal line; box limits indicate the 25th and 75th percentiles, and whiskers show the minimum and maximum values (excluding outliers). Jittered points display the distribution of individual data. Statistically significant differences between pairs of sites are indicated by horizontal brackets (Steel-Dwass post hoc test, *p* < 0.05 *, *p* < 0.01 **, *p* < 0.001 ***). Colors indicate the tissue analyzed: liver (blue), diaphragm (red), and testicles (gray).

**Figure 5:**
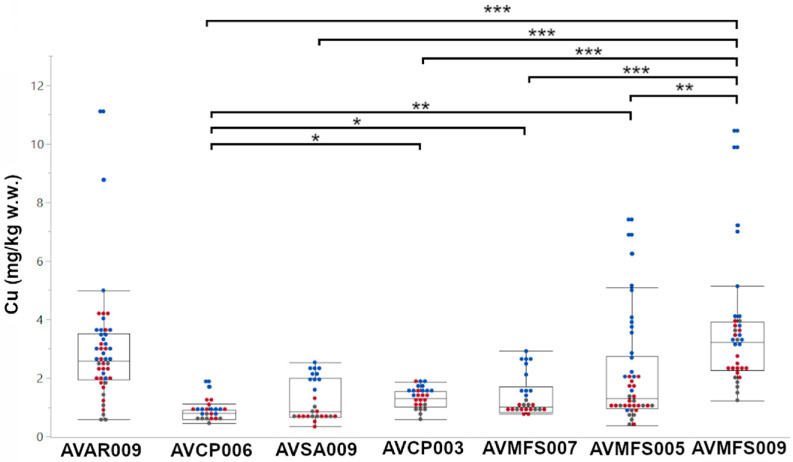
Box-and-whisker plots show the geographical distribution of Cu concentrations (mg/kg w.w.) at sample locations in the province of Avellino. The median is indicated by a horizontal line; box limits represent the 25th and 75th percentiles, and whiskers show the minimum and maximum values (excluding outliers). Jittered points display the distribution of individual data. Statistically significant differences between pairs of sites are marked by horizontal brackets (Steel-Dwass post hoc test, *p* < 0.05 *, *p* < 0.01 **, *p* < 0.001 ***). Colors indicate the tissue analyzed: liver (blue), diaphragm (red), and testicles (gray).

**Figure 6:**
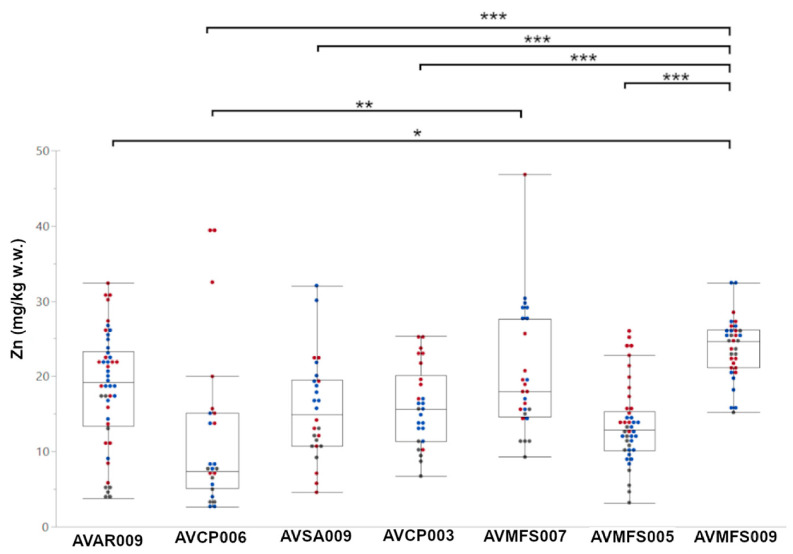
Box-and-whisker plots showing the geographical distribution of Zn concentrations (mg/kg w.w.) at sample locations in the province of Avellino. The median is indicated by a horizontal line; box limits represent the 25th and 75th percentiles, and whiskers show the minimum and maximum values (excluding outliers). Jittered points display the distribution of individual data. Statistically significant differences between pairs of sites are marked by horizontal brackets (Steel-Dwass post hoc test, *p* < 0.05 *, *p* < 0.01 **, *p* < 0.001 ***). Colors indicate the tissue analyzed: liver (blue), diaphragm (red), and testicles (gray).

**Figure 7:**
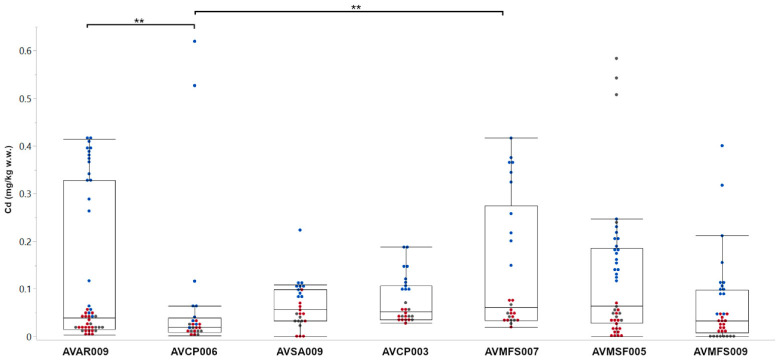
Box-and-whisker plots showing the geographical distribution of Cd concentrations (mg/kg w.w.) at sample locations in the province of Avellino. The median is indicated by a horizontal line; box limits represent the 25th and 75th percentiles, and whiskers show the minimum and maximum values (excluding outliers). Jittered points display the distribution of individual data. Statistically significant differences between pairs of sites are marked by horizontal brackets Steel-Dwass post hoc test, *p* < 0.01 **). Colors indicate the tissue analyzed: liver (blue), diaphragm (red), and testicles (gray).

**Figure 8:**
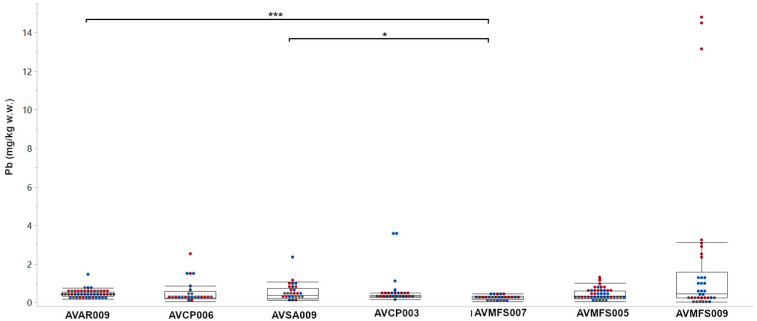
Box-and-whisker plots showing the geographical distribution of Pb concentrations (mg/kg w.w.) at sample locations in the province of Avellino. The median is indicated by a horizontal line; box limits represent the 25th and 75th percentiles, and whiskers show the minimum and maximum values (excluding outliers). Jittered points display the distribution of individual data. Statistically significant differences between pairs of sites are marked by horizontal brackets (Steel-Dwass post hoc test, *p* < 0.05 *, *p* < 0.001 ***). Colors indicate the tissue analyzed: liver (blue), diaphragm (red), and testicles (gray).

**Figure 9:**
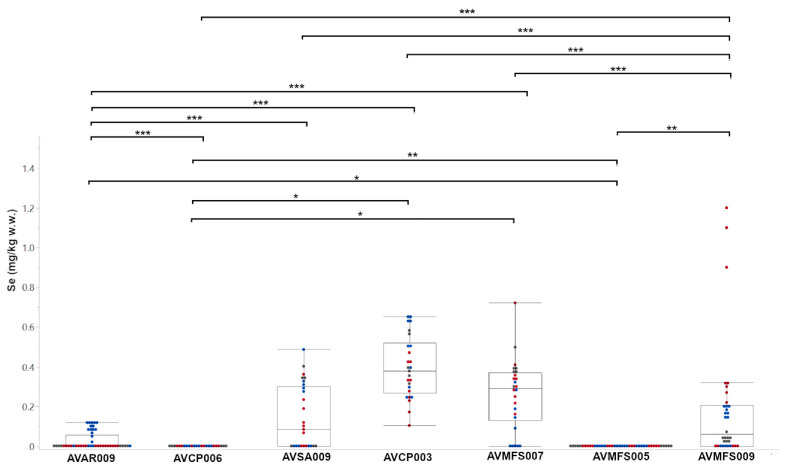
Box-and-whisker plots showing the geographical distribution of Se concentrations (mg/kg w.w.) at sample locations in the province of Avellino. The median is indicated by a horizontal line; box limits represent the 25th and 75th percentiles, and whiskers show the minimum and maximum values (excluding outliers). Jittered points display the distribution of individual data. Statistically significant differences between pairs of sites are marked by horizontal brackets (Steel-Dwass post hoc test, *p* < 0.05 *, *p* < 0.01 **, *p* < 0.001 ***). Colors indicate the tissue analyzed: liver (blue), diaphragm (red), and testicles (gray).

Geographical analysis of the sampling sites revealed significant geochemical differences for all elements studied (*p* < 0.0001, Kruskal–Wallis) (Figure 4, Figure 5, Figure 6, Figure 7, Figure 8 and Figure 9). The AVMFS009 district was the primary location for Cu and Zn accumulation, with significantly higher excesses than AVCP006, AVCP003, AVMFS007 and AVSA009. Compared to AVCP006, AVAR009 also showed very high Cu and Zn contents. AVCP003 was identified as a key hotspot for As, exhibiting substantial excesses compared to AVCP006, AVAR009, and AVMFS007, and much higher levels than AVSA009. The As concentrations detected in AVMFS009 were much higher than those found in AVMFS005 and AVCP006. Significant peaks for Cd were observed only in AVMFS009 and AVAR009 compared to AVCP006. Se concentrations in AVCP003 and AVMFS009 were much higher than those in AVAR009 and AVMFS005. The distribution of Pb showed less geographical variability, but AVMFS005 and AVMFS009 had significantly higher levels than the mountain areas.

## 4. Discussion

The results of this pilot study provide a comprehensive overview of the bioaccumulation patterns of essential and non-essential trace elements in wild boars (*Sus scrofa*) from the province of Avellino. When evaluating trace element bioaccumulation in a geographically complex territory such as the Italian peninsula, macro-regional extrapolations are inherently flawed. Because of the pronounced latitudinal and geomorphological gradients in the country, wild boar populations exhibit high ecological and phenotypic plasticity. Populations adapted to the continental, alluvial plain, or alpine ecosystems of northern Italy experience fundamentally different trophic resources, metabolic pressures, and baseline environmental exposures compared with populations in southern Italy. Our study specifically addresses the lack of data on the unique Mediterranean, pyroclastic-derived environments of the southern Apennines [24]. To our knowledge, this is the first study to simultaneously compare levels of As, Cd, Cu, Se, Zn, and Pb in liver, diaphragm, and testicular tissues across three distinct environmental gradients within this specific bioregion. Characterizing this ecological niche, where volcanic soils naturally enrich the food web with specific geogenic metalloids such as As and Se, is crucial because it provides a highly localized toxicological baseline that cannot be replicated by or directly compared with existing literature from geographically distant Italian macro-regions. Principal component analysis and tissue-specific correlation analysis revealed complex dynamics between environmental exposure and physiological homeostasis. Wild boars (*Sus scrofa*) serve as an important ecological interface and are widely recognized as effective bioindicators of heavy-metal contamination due to their omnivorous diet, broad spatial distribution, and the tendency of metabolically active organs to accumulate heavy metals and metalloids as a result of their central role in detoxification and metabolic regulation [25]. The significant elemental compartmentalization observed in this study reflects the different biological functions and toxicokinetics of the analytes. Zn was the most abundant element in all matrices, showing a high affinity for muscle tissue (diaphragm) due to its essential roles in muscle contraction, enzymatic activity, and antioxidant defense [26,27]. No significant changes in Zn levels in the liver and diaphragm were observed, suggesting highly saturated, regulated physiological pools in these tissues. Conversely, the lower level in testicular tissue is likely due to more stringent regulatory systems designed to protect reproductive function for systemic fluctuation [28]. These trends are highly consistent with those observed in Spain, Sweden, and Hungary, where Zn is demonstrated to be dominant in skeletal muscle [29,30,31].

The highest concentrations of Cu were found in the liver, significantly those in the diaphragm and testicles, consistent with the liver’s major role in Cu homeostasis, ceruloplasmin synthesis, and storage [32]. Cellular defense mechanisms against toxic metals are closely linked to essential elements. Our correlation analysis revealed strong synergistic covariation among hepatic Cu, Zn, and Cd (ρ = 0.2408 and ρ = 0.2265, respectively). Intracellular accumulation of toxic xenobiotics such as Cd induces metallothioneins (MTs), low-molecular-weight, cysteine-rich proteins. MTs sequester free toxic metal ions to prevent cellular damage; however, because of their biochemical structure, they simultaneously bind and homeostatically regulate essential trace metals, particularly Cu and Zn [33,34]. This shared metabolic binding pathway explains the strong synergistic covariation observed in wild boars from Avellino and is consistent with hepatic profiles documented in Central and Eastern European populations [35,36].

Pb was the most heterogeneously distributed element, showing significantly higher variability in the diaphragm. Such sporadic increases indicate acute or episodic exposure, higl indicative of the ingestion of contaminated soil particles or fragments from traditional Pb-based ammunition. The fragmentation of Pb bullets on impact is a known route of exposure in free-ranging ungulates, often leading to localized, very high concentrations in muscular tissues [37,38,39]. No marked differences in baseline level were noted between the liver and diaphragm, indicating that both tissues are important compartments for Pb deposition in chronic low exposure, while much lower amounts in testicular tissue support the protective role of the blood-testicles barrier [40].

In contrast, Cd showed a strong tropism for the liver, with concentrations significantly higher than in other tissues (*p* < 0.0001). This finding supports the hypothesis of chronic Cd exposure, as the liver is the primary site of Cd accumulation and long-term storage [41].

An interesting finding of our study is the statistical discrepancy observed for hepatic Cd accumulation. The Kruskal–Wallis test showed significant age-dependent differences (*p* = 0.0231), whereas the more conservative Steel-Dwass post hoc test did not identify specific significant differences between pairs of groups. From a methodological perspective, this discrepancy is primarily related to the conservative nature of the nonparametric post hoc test, combined with the relatively small sample size of the older cohort (Senior, *n* = 8). Consequently, given the opportunistic nature of the convenience sampling and the limited size of the Senior group, this statistical pattern cannot be definitively interpreted as a clear biological pathway. Although Cd is known to be a cumulative toxicant with a long biological half-life [42,43], these results should be treated strictly as a preliminary descriptive trend rather than as robust evidence of progressive bioaccumulation, underscoring the need for validation in a larger, more balanced population sample.

The presence of Cd in wild boars in the province of Avellino is most likely related to diffuse, non-point environmental sources. There are no large industrial facilities, but several human activities can release Cd into the local environment: prolonged use of phosphate fertilizers, vehicle emissions along main valley routes, domestic and agricultural combustion processes, and historical atmospheric deposition. The high persistence of Cd in soils and its increased bioavailability in the acidic, volcanic-derived substrates typical of this area favor its transfer to plants, fungi, and soil invertebrates. While foraging by rooting, wild boars ingest soil, roots, and earthworms, making them especially susceptible to the bioaccumulation of this metal. The levels of substances found in Avellino are probably associated with chronic exposure to low but pervasive environmental contamination, not with emissions from specific industrial point sources, as hypothesized in other Italian regions [25,40,44,45].

In contrast to the generally low and stable levels of selenium (Se) and arsenic (As) across the province, the spatial analysis revealed highly localized, distinct environmental signatures that point to specific geogenic and anthropogenic pathways. The district of Montella (AVCP003) emerged as a highly specific hotspot for As and Se. Located within the Picentini Mountains, Montella is characterized by acidic, volcanically derived soils and is widely known for intensive chestnut cultivation. In Campania, pyroclastic substrates are naturally enriched in geogenic arsenic. The acidic nature of these volcanic soils significantly enhances the environmental bioavailability and mobility of geogenic As and Se, facilitating their transfer from soil to forest vegetation and hypogeous fungi, which are notorious hyperaccumulators of metalloids. Because wild boars in Montella forage extensively on chestnuts, acorns, and roots, this trophic pathway explains the highly localized and elevated As and Se levels compared to other agrarian, clay-dominated districts such as Bonito/Grottaminarda (AVAR009) or Chiusano S.D. (AVCP006).

By contrast, the district of Venticano/Pietradefusi (AVMFS009) emerged as a major multi-element hotspot for Cu, Zn, and Cd. Rather than indicating industrial point sources, this spatial signature is closely linked to intensive agricultural activities, particularly viticulture, which is prominent in the middle Calore Valley. Historically, these crops have received intensive applications of copper-based fungicides (such as copper sulfate) and phosphate fertilizers. Phosphate fertilizers are well-documented vectors of diffuse environmental cadmium contamination, as raw rock phosphate naturally contains varying levels of Cd as an impurity, which is subsequently transferred to agricultural soils and bioaccumulated by rooting wild boars. Furthermore, AVMFS009 is crossed by major regional highways (A16 Napoli–Canosa), suggesting that traffic-related emissions, specifically tire wear (a major source of environmental Zn and Cd) and historical exhaust deposits, contribute to this localized multi-element burden.

Principal component analysis (Figure 3), focusing exclusively on fully quantified elements (Pb, Cd, Cu, and Zn), accounted for 67.7% of the total variance and clearly revealed spatial segregation of metabolic (liver) and muscular (diaphragm) signals. In the loading plot (Figure 3B), Cd, Zn, and Cu emerged as the main drivers along the PC1 axis, highlighting tissue-specific metabolic pathways, while the vertical distribution of the Pb vector along PC2 reflected its independent bioaccumulation profile, particularly separating the distinct tissue matrices.

This continuous bioaccumulation model is supported by our Spearman correlation results, which revealed highly tissue-specific kinetics. In skeletal muscle (diaphragm), Zn concentrations showed a significant positive correlation with body weight (ρ = 0.2308, *p* = 0.0315), reflecting the essential metabolic role of this micronutrient. Zinc is a cofactor for multiple enzyme systems that regulate cell division, protein synthesis, and muscle hypertrophy, which explains its continuous accumulation in muscle tissue in parallel with physical growth [46].

Surprisingly, Cd concentrations in the testicles of males (*n* = 50) showed a highly significant negative correlation with body weight (ρ = −0.3442, *p* = 0.0144). This downward trend is consistent with the physiological phenomenon of “growth dilution” [47]. During sexual maturation, wild boar testicles undergo massive hypertrophy, characterized by a rapid increase in gonadal mass, testicular volume, and seminal fluid production. Consequently, although the absolute amount of Cd in the organ remains constant or increases slightly, its concentration per unit mass decreases dramatically in mature, heavier males compared with the small, undeveloped gonads of younger specimens.

Finally, the consistently observed highly significant positive correlations between Cd, Zn, and Cu in liver tissue reflect well-documented cellular defense mechanisms. The intracellular accumulation of toxic xenobiotics such as Cd triggers the induction of low-molecular-weight, cysteine-rich proteins known as metallothioneins (MTs). These proteins sequester free toxic metal ions and prevent cellular damage. However, due to their biochemical structure, MTs simultaneously bind essential trace metals, particularly Cu and Zn. This shared metabolic binding pathway explains the strong synergistic covariation observed between hepatic Cu, Zn, and Cd, highlighting a coordinated homeostatic response to environmental metal exposure in the sampled wild boar population [48].

Conversely, in gonadal tissue (testicles), the highly significant negative correlation observed between Zn and Pb (ρ = −0.3709, *p* = 0.0080) has critical reproductive toxicological implications. Pb is known to exert gonadotoxicity by directly competing with essential divalent cations, primarily Zn, for binding sites on regulatory proteins and metalloenzymes. In the testicles, zinc is an indispensable micronutrient that maintains chromatin structural integrity, regulates follicle-stimulating hormone (FSH) and luteinizing hormone (LH) receptors, and serves as a cofactor for key enzymes governing spermatogenesis, such as alkaline phosphatase and carbonic anhydrase. The strong negative correlation suggests that Pb bioaccumulation may competitively displace zinc, leading to localized functional Zn deficiency. This enzymatic inhibition and structural disruption in germ cells can impair sperm motility, trigger premature apoptosis of spermatogonia, and compromise overall reproductive capacity. Furthermore, the co-accumulation of even low levels of toxic elements such as Cd and Pb in the gonads poses an interactive risk of endocrine disruption, highlighting the testicles as a highly sensitive target organ that must be included in comprehensive One Health wildlife health assessments [48].

Regarding sexual dimorphism, we found significant sex-dependent differences (Table 3). Males had significantly higher levels of Pb in the liver (*p* = 0.0002) and Cd in the diaphragm (*p* = 0.0017). Liver levels of Cd and As were higher in females (*p* < 0.05). These differences may be related to different foraging patterns, territory size, or metabolic changes associated with the reproductive cycle (such as pregnancy and lactation) that can influence the mobilization of metals from bone or soft tissue.

To establish a baseline for public health screening, tissue concentrations of Pb and Cd were compared with the maximum regulatory limits (MRLs) established by Commission Regulation (EU) 2024/1756 [22] for domestic pigs [49]. Although a formal quantitative dietary risk assessment (including the calculation of daily intakes and hazard quotients) is beyond the scope of this preliminary pilot study, comparing these concentrations with commercial porcine MRLs provides a standardized metric to evaluate the severity of environmental accumulation in edible tissues. Although median Pb values in the liver and diaphragm were below these thresholds, individual outliers in muscle tissue substantially exceeded the legal safety limits. This finding underscores a critical toxicological concern for consumers of game meat, as the fragmentation of lead ammunition poses localized, highly elevated exposure risks [38,39].

The discrepancy between the median Pb levels and the maximum concentration in the diaphragm suggests acute, localized contamination rather than systemic absorption. Although precise data on the exact distance of each collected sample from the wound channel or the specific ammunition type were not available, this spike is very likely influenced by the hunting method and bullet fragmentation. Traditional lead-based bullets fragment extensively upon impact, dispersing microscopic lead particles along the wound tract and into adjacent tissues such as the diaphragm. If sampling occurs near these areas, or if the bullet directly traverses the thoracic-abdominal boundary, the risk of incorporating ammunition debris into the analyzed matrix increases substantially, which explains the observed extreme outliers.

Regarding Cd, although the median liver concentration remained below the domestic porcine safety threshold of 0.50 mg/kg w.w., the strong hepatic tropism of this toxicant indicates that the liver serves as a major chronic reservoir. Therefore, high dietary game consumption within hunting households that practice self-consumption (“autoconsumo”) could result in substantial cumulative cadmium intake.

## 5. Conclusions

The study provides an integrated evaluation of trace element accumulation in wild boars from Avellino province, revealing distinct tissue-specific accumulation patterns and exposure signatures consistent with diffuse environmental contamination. The liver showed a pronounced affinity for Cd, while Pb was unevenly distributed, with some high levels in the diaphragm, indicating a combination of chronic background exposure and sporadic episodic inputs, such as ingestion of Pb ammunition fragments. Essential elements showed the expected physiological gradients between tissues, with Cu enriched in the liver and Zn dominant in the diaphragm. Se and As were uniformly low in all tissues. Comparison with data from other European and non-European regions suggests that the metal burdens observed in the Avellino population are characteristic of areas affected by nonpoint agricultural, geochemical, and widespread anthropogenic sources, rather than industrial point emissions.

In summary, this study demonstrates that trace element accumulation in biological matrices is governed by a three-way interaction among tissue-specific physiological functions, geographical environmental pressures, and biological factors such as sex. Testicular tissue was excluded from the sexual dimorphism analysis because there is no anatomical equivalent in females, providing a biologically sound framework for comparison. The identification of specific hotspots such as Montella (AVCP003) (driven by geogenic arsenic bioavailability in volcanic soils) and Venticano/Pietradefusi (AVMFS009) (linked to diffuse viticulture inputs and valley traffic) provides essential data for targeted environmental management and highlights the importance of multi-matrix monitoring to capture the full range of bioaccumulation. The results indicate that environmental contamination in the province of Avellino is generally low but geographically widespread, consistent with the volcanic-derived soils and agricultural activities of the region. The presence of localized peaks of As in AVCP003 and a multi-elemental signature in AVMFS009 underscores the need for a site-specific approach to environmental health. Although median concentrations were generally within European Union Maximum Residue Limits (MRLs), the presence of muscle tissue outliers exceeding commercial limits for Pb underscores the need for localized monitoring from a public health surveillance perspective. Because a formal dietary risk assessment was beyond the scope of this study, these data should be interpreted strictly as a preliminary screen of regulatory compliance rather than a definitive quantification of human exposure. Ongoing biomonitoring is recommended to track temporal trends, identify emerging contaminants, and provide baseline data for future One Health dietary risk assessments for human consumers.

## Figures and Tables

**Table 1:** Validation parameters of the ICP-MS method for the determination of essential and non-essential elements. LOD: limit of detection; LOQ: limit of quantification; RSD: relative standard deviation; w.w.: wet weight.

Element	LOD (µg kg^−1^ w.w.)	LOQ (µg kg^−1^ w.w.)	Correlation Coefficient (R^2^)	Recovery (%)	Precision (RSD, %, *n* = 6)
As	0.008	0.027	0.999	96.5	3.2
Cd	0.001	0.003	0.998	98.7	2.8
Cu	0.012	0.040	0.999	101.2	2.4
Pb	0.003	0.010	0.998	98.3	3.6
Se	0.015	0.050	0.998	95.2	4.5
Zn	0.020	0.067	0.999	98.2	2.1

**Table 2:** Descriptive statistics (mean ± SE, median, and range) for Pb, Cd, Cu, Zn, Se, and As concentrations (mg/kg w.w.) in liver, diaphragm, and testicular tissues of wild boars (*Sus scrofa*). Sample sizes: liver (*n* = 87), diaphragm (*n* = 87), testicles (*n* = 50). For heavily left-censored elements (As, Se, and baseline Cd), medians or minimum range boundaries are reported as <0.001 mg/kg (<LOD) to prevent substitution artifacts. Minor approximations in upper range limits result from JMP software graphical scale fitting during distribution analysis.

Element	Organ	N°	Mean ± SE	Median	Range (Min–Max)
	Liver	87	0.55 ± 0.07	0.34	0.07–3.58
Pb (mg/Kg w.w.)	Diaphragm	87	1.07 ± 0.27	0.43	0.07–14.80
	Testicles	50	0.24 ± 0.02	0.25	0.019–0.70
	Liver	87	0.20 ± 0.01	0.15	0.02–0.62
Cd (mg/Kg w.w.)	Diaphragm	87	0.03 ± 0.002	0.03	<0.001–0.09
	Testicles	50	0.06 ± 0.02	0.031	<0.001–0.58
	Liver	87	3.09 ± 0.23	2.58	0.75–11.10
Cu (mg/Kg w.w.)	Diaphragm	87	1.61 ± 0.10	1.15	0.34–4.21
	Testicles	50	1.14 ± 0.11	0.87	0.45–3.89
	Liver	87	17.76 ± 0.77	16.81	2.69–32.43
Zn (mg/Kg w.w.)	Diaphragm	87	19.99 ± 0.82	20.51	4.59–46.81
	Testicles	50	11.61 ± 0.90	11	3.07–26.2
	Liver	87	0.11 ± 0.02	<0.001	<0.001–0.65
Se (mg/Kg w.w.)	Diaphragm	87	0.14 ± 0.02	<0.001	<0.001–1.10
	Testicles	50	0.13 ± 0.03	<0.001	<0.001–0.33
	Liver	87	0.03 ± 0.003	0.03	<0.001–0.08
As (mg/Kg w.w.)	Diaphragm	87	0.02 ± 0.003	<0.001	<0.001–0.09
	Testicles	50	0.013 ± 0.003	<0.001	<0.001–0.04

**Table 3:** Results of the Kruskal–Wallis test (χ^2^) and Steel–Dwass post hoc comparisons between the different biological matrices. The significance of the pairwise comparisons is indicated as follows: *p* > 0.05 ns; *p* < 0.001 ***; *p* < 0.0001 ****.

Element	χ^2^	*p* Value	Liver vs. Diaphragm	Testicles vs. Liver	Testicles vs. Diaphragm
Pb	28.1079	<0.0001	ns	***	****
Cd	123.8754	<0.0001	****	****	ns
Cu	67.7456	<0.0001	****	****	****
Zn	39.1122	<0.0001	ns	****	****
Se	0.2277	0.8924	ns	ns	ns
As	12.6688	0.0018	ns	***	ns

**Table 4:** Comparison of median levels (mg/kg w.w.) between male (*n* = 50) and female (*n* = 37) specimens in liver and diaphragm tissues. Statistical significance was assessed using the Wilcoxon–Mann–Whitney test (*p* < |Z|). Medians for heavily left-censored elements (As and Se) are reported as <0.001 mg/kg (<LOD) to avoid substitution artifacts. Minor approximations in upper range boundaries result from JMP software graphical scaling and automated smoothing. The significance of the pairwise comparisons is indicated as follows: *p* > 0.05 ns; *p* < 0.05 *; *p* < 0.01 **; *p* < 0.001 ***.

Organ	Element	Male *n*° = 50 Median (Min–Max)	Female *n*° = 37 Median (Min–Max)	*p*-Value (Prob > |Z|)	Significance
Diaphragm	Pb	0.48 (0.16–14.8)	0.363 (0.068–2.52)	0.096	ns
	Cd	0.034 (<0.001–0.094)	0.015 (<0.001–0.065)	0.0017	**
	Cu	1.32 (0.34–4.21)	1.12 (0.67–2.60)	0.665	ns
	Zn	21.88 (4.59–39.42)	18.77 (5.77–46.82)	0.0142	*
	Se	0.081 (<0.001–1.2)	<0.001 (<0.001–0.72)	0.0097	**
	As	0.0195 (<0.001–0.091)	<0.001 (<0.001–0.96)	0.035	*
Liver	Pb	0.43 (0.073–3.58)	0.24 (0.11–1.46)	0.0002	***
	Cd	0.11 (0.018–0.42)	0.22 (0.064–0.62)	0.0048	**
	Cu	2.58 (0.75–4.05)	2.57 (0.87–11.1)	0.199	ns
	Zn	16.4 (2.7–32.4)	16.9 (7.4–32.0)	0.379	ns
	Se	<0.001 (<0.001–0.50)	0.021 (<0.001–0.65)	0.495	ns
	As	<0.001 (<0.001–0.097)	0.051 (<0.001–0.076)	0.0144	*

**Table 5:** Heavy metal and trace element concentrations (mg/kg w.w.) in diaphragm and liver tissues of wild boars (*n* = 87) across biological age classes (Young, Adult, Senior), including Kruskal–Wallis test statistics (χ^2^) and post hoc pairwise comparison results. Values are expressed as medians and interquartile ranges (25th–75th percentiles). Significant differences (*p* < 0.05) are highlighted in bold and marked with an asterisk (*). “ns” indicates non-significant differences.

Organ	Element	Young *n*° = 33 Median (Min–Max)	Adult *n*° = 46 Median (Min–Max)	Senior *n*° = 8 Median (Min–Max)	*p*-Value (Prob > ChiSq)	Steel–Dwass Test
Diaphragm	Pb	0.35 (0.24–0.51)	0.51 (0.27–0.66)	0.50 (0.25–2.8)	0.237	ns
	Cd	0.03 (0.01–0.04)	0.03 (0.01–0.04)	0.02 (0.01–0.04)	0.706	ns
	Cu	1.27 (0.93–2.26)	1.12 (0.91–2.04)	1.42 (0.51–3.37)	0.815	ns
	Zn	21.3 (16.3–23.4)	18.9 (14.8–25.2)	21.1 (8.3–24.9)	0.925	ns
Liver	Pb	0.36 (0.21–0.95)	0.35 (0.2–0.52)	0.31 (0.18–1.2)	0.632	ns
	Cd	0.14 (0.1–0.2)	0.2 (0.11–0.37)	0.1 (0.03–0.18)	0.0231 *	ns
	Cu	2.2 (1.6–4.0)	2.6 (1.9–3.6)	2.3 (1.1–3.1)	0.456	ns
	Zn	16.8 (13.8–25.6)	17.7 (12.1–22.5)	14.9 (9.8–17.5)	0.375	ns

**Table 6:** Double-entry Spearman’s rank correlation matrix (ρ) analyzing the relationships between individual body weight (kg) and trace element concentrations (Pb, Cd, Cu, Zn) in diaphragm and liver tissues (*n* = 87) and in testicular tissues (*n* = 50) of wild boars. Statistically significant Spearman’s rank correlation coefficients (ρ) are highlighted in bold. Asterisks indicate the level of statistical significance: * *p* < 0.05; ** *p* < 0.01; *** *p* < 0.001.

Organ		Weight (kg)	Pb	Cd	Cu	Zn
Diaphragm	Weight (kg)	1.000	0.0139	−0.0262	0.1436	0.2308 *
	Pb	0.0139	1.000	0.2911 **	0.3122 **	−0.0066
	Cd	−0.0262	0.2911 **	1.000	−0.0218	0.0729
	Cu	0.1436	0.3122 **	−0.0218	1.000	0.5469 ***
	Zn	0.2308 *	0.0066	0.0729	0.5469 ***	1.000
Liver	Weight (kg)	1.000	−0.0126	0.0951	0.0620	0.0861
	Pb	−0.0126	1.000	−0.1983	0.1649	0.0747
	Cd	0.0951	0.1983	1.000	0.2408 *	0.2265 *
	Cu	0.0620	−0.1983	0.2408 *	1.000	0.4251 ***
	Zn	0.0861	0.0747	0.2265 *	0.4251 ***	1.000
Testicles	Weight (kg)	1.000	−0.1259	−0.3442 *	−0.0219	−0.1650
	Pb	−0.1259	1.000	−0.0308	−0.1418	−0.3709 **
	Cd	−0.3442 *	−0.0308	1.000	−0.2572	−0.2336
	Cu	−0.0219	−0.1418	−0.2572	1.000	0.7564 ***
	Zn	−0.1650	−0.3709 **	−0.2336	0.7564 ***	1.000

## Data Availability

The original contributions presented in this study are included in the article. Further inquiries can be directed to the corresponding authors.

## Abbreviations

The following abbreviations are used in this manuscript:

Cd	Cadmium
As	Arsenic
Pb	Lead
Se	Selenium
Zn	Zinc
Cu	Copper
